# Telling the Truth for Fear of the “Cop”

**Published:** 1891-07

**Authors:** S. H. Preston


### TELLING THE TRUTH FROM FEAR OF THE "COP."

BY S. H. PRESTON.

" Do you know what an oath is?" inquired Coroner Hanly the other day of an Italian lad, in an inquest case of murder.

" No, sir," replied the bright boy, "I never swear."

" Do you know what will become of you if you tell a lie?"

" Yes, sir; you'd tell the cop to get me." The Coroner then administered the oath.

It is no longer necessary to believe in God and future punishment for perjury for one who thinks the "cop" will get him if he lies. Probably a boy believing his father would beat him if he did not tell the truth, would be qualified to take the oath before Coroner Hanly.

But the Coroner is not without a precedent in this matter of juvenile swearing. A boy in charge of the Christian Brothers was called as a witness in a Philadelphia court.

"If you should swear to a lie here, who is it that would surely know it?" solemnly asked the judge.

" The Brothers," promptly replied the boy.

" But who is it who would punish you for it?"

" The Brothers. They would beat me."

" But who else would punish you?"

" Me pap."

" But isn't there some one else who would know if you told a lie?"

" Yes, sir; Mr. Jenkins," (one of the counsel who had talked with him.)

" But," persisted the judge, "isn't there a Great Being above who punishes bad boys who lie?"

"O, and is it God yez is askin' me about?" And he was sworn.

The administration of the oath, even to adults, especially in our city criminal courts, is not calculated to convert a constitutional liar into a George Washington. "U-sholmly-swar-ell-troot-nat-ig-ut-troot-ull-troot-shelp-u-got-'is-de-buk," mumbles the judicial mouthpiece as he lifts to the lips of the witness a battered book, stained with smacks from scores of tobacco-spitting bums and unclean creatures of the slums. Physicians have found that infectious diseases are communicated in this manner; and a particular person would have some sanitary scruples, if not of conscience, against kissing the lip-soiled volume.

It is absurd to suppose that any form of oath will deter folks from swearing falsely when prompted to do so by considerations of pecuniary gain or personal safety. Nothing will prevent them but the prospect of incurring the legal penalty. If they tell the truth it is from fear of punishment for perjury. And it matters not whether the foundation of this fear be a boy's belief in a parent who will beat him, a "cop" who will arrest him, or a God who will send his soul to hell for false swearing.

The man of conscience speaks the truth under all circumstances, whether sworn or not. He acquires a reputation for veracity among all his acquaintances. They accept his statements without the formality of an oath. Thus, in the preliminary inquiry as to the competency of a witness, the question is asked, What is his reputation for truth and veracity ? When Mr. Doe was asked this question about his neighbor Roe, he answered, "His reputation for truth is all right, but there is a good deal of talk about his veracity." "What do you understand by veracity ?" asked the judge. "Why, that he is—rather rough among the wimmin."

Uncle Uriah was witness in a case in which the credibility of his neighbor Norton was called in question. "If Mr. Norton should swear to something he saw, would you believe it ?" asked counsel. "Wall, yes, I guess I would—if I knew it was so." "From what you know of Mr. Norton would you believe him if he was sworn as a witness ?" interposed the judge. "'Fi was you I wouldn't swear him," responded Uncle Uriah.

A notorious, deliberate liar is not believed, even though he kiss the Bible and swear by all that is sacred. So that it is just as well not to swear him. A just and conscientious man will tell the truth on all occasions, whether under oath or not. So that there is no need to swear such a man.

If it be held that folks only tell the truth through fear of punishment for falsehood, then the boy that thinks the "cop" will take him to the Tombs for telling a lie is as worthy a witness as the man.

